# UV-induced Spectral Shift and Protonation of DNA Fluorescent Dye Hoechst 33258

**DOI:** 10.1007/s10895-014-1468-y

**Published:** 2014-10-14

**Authors:** Dominika Żurek-Biesiada, Piotr Waligórski, Jurek W. Dobrucki

**Affiliations:** 1 Division of Cell Biophysics, Faculty of Biochemistry, Biophysics and Biotechnology, Jagiellonian University, ul. Gronostajowa 7, 30-387 Kraków, Poland; 2 The Franciszek Górski Institute of Plant Physiology, Polish Academy of Sciences, ul. Niezapominajek 21, 30-239 Kraków, Poland

**Keywords:** Photoconversion, Hoechst, DNA, Super-resolution microscopy

## Abstract

DNA-bound Hoechst 33258 is readily excited with UV light and emits blue fluorescence, however, upon exposure to UV, the dye undergoes photobleaching as well as photoconversion to a blue-excited green-emitting form. We demonstrate that the UV-generated green-emitting form of Hoechst 33258 exhibits spectral properties very similar to the form of the dye that can be obtained by subjecting it to an acidic environment (pH 0.5–3.0). We also demonstrate that exposure of Hoechst 33258 to UV light (or hydrogen peroxide) leads to generation of the protonated (1+, 2+, 3+ and possibly the 4+) forms of the dye. Photoconversion of Hoechst 33258 has recently been exploited in single molecule localisation microscopy, thus understanding photophysics of this process can facilitate further development of high resolution optical imaging.

## Introduction

Hoechst 33258 is a common fluorescent dye used for staining and visualisation of DNA by fluorescence wide field and confocal microscopy. DNA-bound Hoechst 33258 is readily excited with UV and emits fluorescence in the blue region of the visible light spectrum (exc./em. maxima 355/465 nm). We have recently reported that, upon excitation with UV, Hoechst 33258 undergoes photoconversion [1]. Although a typical loss of fluorescence (photobleaching) was observed, apparently a fraction of the blue-emitting dye molecules did not lose their ability to fluoresce, but were converted to a blue-excited, green-emitting form. We have also demonstrated that the amount of the photoproduct is proportional to the dose of exciting light delivered, and that photoconversion does not require the presence of DNA or water [1]. Preliminary mass spectrometry data suggested that the observed spectral changes were associated with UV-induced protonation of Hoechst molecules [1].

**Fig. 1:**
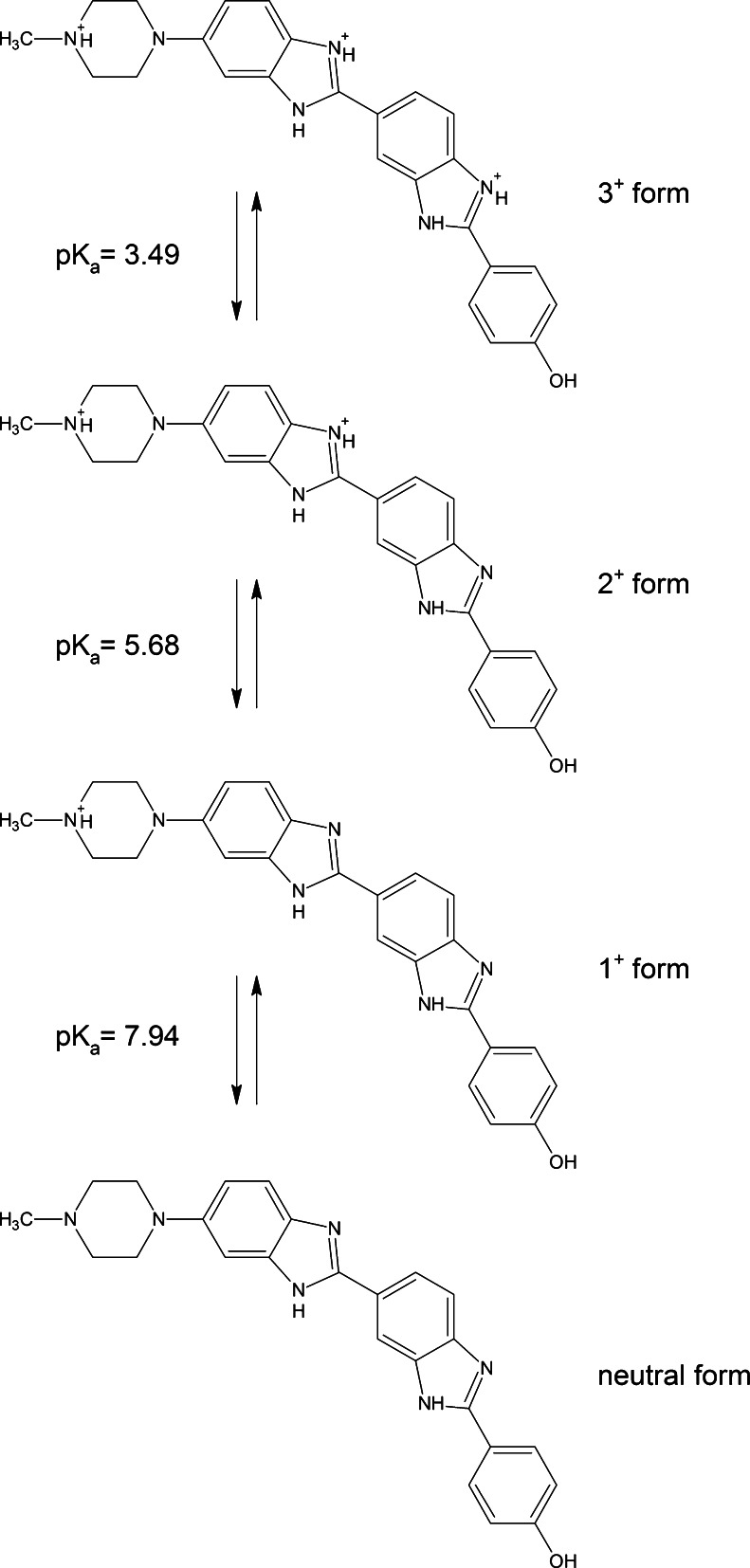
The sequence of protonation of Hoechst 33258 and pKa values, based on Ladinig et al. 2005. Note that the structures proposed here do not include a number of other possible forms, for instance the neutral form, which contains one negative and one positive charge, as described in Aleman et al. 2005, or the 3+ form with protons on different nitrogen atoms

Protonation of Hoechst occurring in solutions of low pH has been described in several reports [2–6]. The hypothetical protonated forms of Hoechst 33258, as described in [3], are redrawn in Fig. 1. To our knowledge, changes of spectral properties of Hoechst that accompany single, double and triple protonation have not been described in detail so far. It is known, however, that the quantum yield of the fluorescence of Hoechst increases 20-fold upon a shift of pH from neutral to 4.5, and falls 80-fold when pH decreases from 4.5 to 1.5 [5]. Based on the available photophysical data one may postulate that it is a protonated form of DNA-bound Hoechst that contributes predominantly to the blue emission typically observed in fluorescence microscopy images of cells maintained in neutral pH. In fact it is known that the blue signals of Hoechst in cell nuclei are more intense if the mounting medium has a low rather than neutral pH.

So far the green emission of photoconverted Hoechst 33258 molecules has been recognised as a nuisance in multicolor fluorescence microscopy, however our recent findings suggest that it can be exploited in super-resolution microscopy [7]. The key to a successful use of the phenomenon of photoconversion of this dye is an understanding of the photophysics of this process. Therefore, in this report we focus our attention on photophysical phenomena underlying the spectral changes induced by UV excitation of Hoechst 33258. We demonstrate that the UV-generated green-emitting form (or forms) of Hoechst 33258 exhibit the same spectral properties as the forms of the dye that can be obtained by subjecting it to a highly acidic environment (pH 0.5–3.0). By using mass spectrometry and spectrofluorimetry we demonstrate that exposing Hoechst 33258 to UV light (or hydrogen peroxide) leads to generation of several protonated forms of the dye. While the 1+, 2+ and 3+ forms of the dye appear to exhibit strong affinity to DNA (binding to RNA is very weak), the protonated form which exists in pH 0.5 (presumably the 4+) exhibits affinity to RNA.

## Materials and Methods

### Cells

MSU 1.1 human fibroblasts [8] were grown in Dulbecco’s Modified Eagles Medium (Sigma-Aldrich, Poland) supplemented with 10 % fetal bovine serum, penicillin (50 units/ml) and streptomycin (50 μg/ml), in tissue culture Petri dishes (Techno Plastic Products AG, Switzerland) at 37 °C, in a humidified atmosphere of 95 % air and 5 % CO_2_. Cells were grown on 20 mm-diameter coverslips (Menzel-Gläser, Germany).

Cells were fixed with 4 % formaldehyde (Electron Microscopy Sciences, USA) using standard formaldehyde fixation protocol and subsequently stored in 1 % formaldehyde solution for 1–2 weeks prior to the experiment.

### Cell Staining

Preparations of fixed cells were rinsed three times with PBS, permeabilised with 70 % ethanol (30 s) and incubated with a solution of Hoechst 33258 [9–11] (2 μg/ml; Sigma-Aldrich, Poland) for 30 min at room temperature (RT).

### Acquisition of Fluorescence Images

Images were recorded using Leica TCS SP5 confocal microscope (Leica Microsystems, Germany), equipped with a 63× 1.4 NA oil immersion lens. 512 × 512 pixel images (field of view 145 × 145 μm) were recorded. PMT gain was set at 860 V; confocal iris was set at 1 Airy disk. When studying the process of UV-excited dye photoconversion, coverslips with the attached fixed cells were mounted in custom made steel holders, placed in a microscope stage and imaged at RT in PBS or solutions of various pH (fixed cells). For excitation a 100 mW Ar ion gas (458 nm) and 405 nm diode (3 mW output) lasers were used. For imaging Hoechst green-emitting products, the intensity of light in the 458 laser line was adjusted to 0,95 mW. Light intensities were measured with a FieldMaxII Laser Power Meter (Coherent, USA). Fluorescent dyes were photoconverted using UV emitted by a Leica EL6000 mercury metal halide lamp, which passed through a 360/40 nm filter (11 mW).

### Data Analysis

Images were analysed and processed using LAS AF Lite (Leica Microsystems, Germany) and MacBiophotonics ImageJ (http://rsbweb.nih.gov/ij/) software. The displayed images were not manipulated beyond adjusting the γ-function, as noted in the figure legends.

### Mass Spectrometry (MS)

Mass spectra were collected with Agilent Technologies 6410 Triple Quad LC/MS mass spectrometer equipped with Electrospray Interface (ESI). Drying gas temperature was set at 350 °C with flow 12 l/min. Nebuliser pressure was 35 psi, capillary voltage 3,000 V. Mass spectra were collected in positive ion mode in the m/z range from 50 to 1,000. Various fragmentor voltages were checked and 10 V was selected as the best one; this provided the highest signal and the lowest fragmentation of the investigated molecules. 5 μl of each sample was injected through an automatic autosampler, a continuous flow of 1:1 mixture of A and B was set, where (A) was water with 0.01 % formic acid and (B) was acetonitryle/methanol (1:1 v/v). The flow was forced with the HPLC pump (Agilent Technologies 1260 series) with flow rate of 0.1 ml/min. The concentration of Hoechst used in mass-spectrometry experiments was 25 μM. Mass spectra were collected and analysed at the point of the highest signal using Mass Hunter software (Agilent Technologies).

**Fig. 2:**
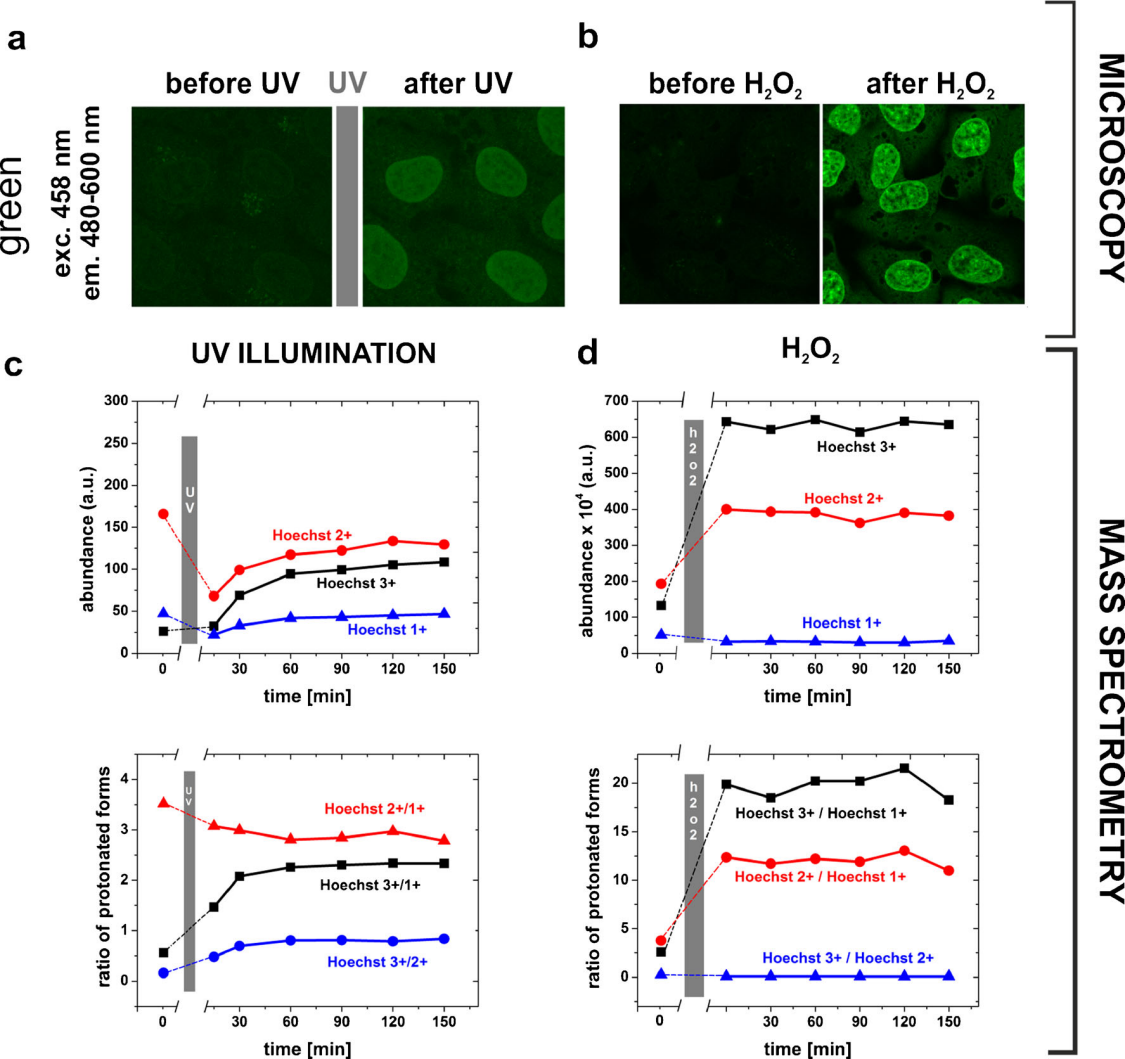
Conversion of Hoechst 33258 to green-emitting forms induced by exposure to UV or hydrogen peroxide in fixed MSU 1.1 fibroblasts stained with Hoechst 33258 (2 μg/ml), detected by fluorescence confocal microscopy (a,b) and mass spectrometry (c,d). **a**, **b** – Images of the green emission of Hoechst-stained cells prior and following exposure to UV (a) or hydrogen peroxide (30 %) (b) Excitation: 458 nm, emission: 480–600 nm (as demonstrated previously [1], Figs. 2–4 and 6). The γ-function of the images in (a) was set to 0.5. **c**, **d** – Conversion and stability in time of the protonated forms of Hoechst 33258 measured by mass spectrometry. Three peaks corresponding to m/z = 142.4 (Hoechst 3+), m/z = 213.1 (Hoechst 2+) and m/z = 425.2 (Hoechst 1+) were prominent in MS spectra. Abundances of the protonated forms of Hoechst 33258 measured after illuminating the sample with UV or after subjecting it to 4 % H_2_O_2_ confirm the induction of protonation and indicate that the protonated forms are stable in solution

For time course mass spectrometry measurements (Fig. 2c,d) Hoechst 33258 solutions were placed in a small glass container (the total volume of 2 ml) and 60 μl of the UV-illuminated solution or 1 ml for the solution of Hoechst treated with hydrogen peroxide was used. An automatic autosampler collected 5 μl of the dye solution and injected into the mass spectrometer at 30 min intervals. The photoconverted forms of Hoechst 33258 needed for these MS analyses were produced by illuminating a solution of the dye with UV on a microscope stage (Nikon Optiphot, equipped with an HBO mercury arc lamp, 330–380 emission filter and a 100x NA 1.3 objective lens). A solution of Hoechst 33258 was prepared (25 μM) and subsequently the samples of approximately 2–3 μl were placed on a microscope slide and each one was illuminated with UV for 30 s. Subsequently the drops of the illuminated dye solution were collected, pooled together and placed in a small glass container, from which the autosampler collected the samples for mass spectrometry analyses.

### Solutions

Solutions of various pH (0.5–12.5) were prepared using distilled water with pH adjusted with NaOH and HCl or HCOOH and NH_3_.

### Spectrofluorimetry

Hoechst 33258 (10 μM) was dissolved in solutions of pH 0.5, 2.0, 3.0, 4.0, 7.4 and 11.5. Samples were placed in quartz cuvettes and analysed using Perkin Elmer LS50B spectrofluorimeter.

## Results

### UV- and H_2_O_2_-induced Generation of Protonated Forms of Hoechst 33258

We have recently described spectral characteristics of Hoechst 33258 after illumination with UV, as well as after subjecting it to highly oxidative conditions (30 % H_2_O_2_) [1]. In both cases the dye was converted to forms that were excited with blue light, and emitted green fluorescence (Fig. 2a,b). In order to verify and extend the preliminary studies of the chemical nature of these green-emitting forms, we performed mass spectrometry analyses of solutions of Hoechst 33258 subjected to UV or oxidising conditions. In samples containing the original and the photoconverted forms of the dye three peaks were detected by mass spectrometry. They correspond to three protonated forms of the dye: a mono-, di- and tri-cation, with their respective mass-to-charge ratios: 425.2, 213.1, 142.4 (examples of MS spectra are given below). A similar result was obtained when Hoechst 33258 was exposed to hydrogen peroxide (Fig. 2b).

Mass spectrometry analysis demonstrated that exposure to UV resulted in a significant decrease of the abundance of the detectable di-cation, a small decrease of the mono-cation, and an increase of the tri-cation (Fig. 2c). Analysis of the ratios between the abundances of different protonated forms indicate that the ratio of 3+/1+ increased, while the 2+/1+ ratio decreased significantly. It is important to note that MS determinations of the abundance of the protonated forms prior and after the UV exposure was done on 2 separate samples, thus the accuracy of a direct comparison between these abundances is limited. Moreover, the total amount of detectable photoproducts generated by UV is fairly limited too, due to their photobleaching which occurs upon exposure to UV. Thus, the actual abundance of the photoconverted forms detected by MS, following exposure to UV, was dependent not only on their generation rate, but on their susceptibility to photobleaching as well. Nevertheless, these data hint at a possibility of UV inducing a single, double and triple protonation of the neutral form and suggest that these forms of the dye are quite stable in solution. A slight increase of the abundance of the 2+ and 3+ form seen in Fig. 2c is likely a result of a slow evaporation of the sample which was maintained in the mass spectrometer. The volume of this sample was only approx. 60 μl, since it was not practical to generate the Hoechst photoproducts by UV in larger quantities (see Materials and Methods). Small amounts of the solution were taken from this sample at time intervals for MS analysis. The evaporation led to an increase of the concentration of the protonated forms in the sample which was maintained in the spectrometer. The volume of the sample in which Hoechst 33258 was exposed to hydrogen peroxide was much larger (1 ml), however the exposed surface was the same as in the small sample treated by UV, thus evaporation had a negligible effect on the concentration of the protonated forms of the dye in this case (Fig. 2d).

Unfortunately mass spectrometry analysis does not provide information about the abundance of the neutral form, therefore the yield of its photoconversion into a single protonated form remains unknown.

Exposing Hoechst to oxidising conditions resulted in a dramatic increase of the abundance of the 3+ and 2+ forms (Fig. 2d). The ratio of 2+/1+ and 3+/1+ increased significantly. The abundances and the ratios between different forms remained unaltered for 2.5 h after adding hydrogen peroxide to the sample (Fig. 2d). This observation is consistent with a notion that exposure to H_2_O_2_ induced protonation of the Hoechst molecule, resulting in high levels of di- and tri-cation. All the protonated forms appeared quite stable in water. It remains unknown, however, if oxidation also led to destruction of some of the dye molecules, for instance to breakage of the rings or fragmentation of the molecule. Although the peaks representing degradation products were not seen on mass spectra, such a possibility cannot be excluded entirely, since small fragments of less than 100 mass-to-charge ratio were not detectable.

In our previous report we demonstrated that the equilibrium between the photoconverted, green-emitting form of the dye, and the original, blue-emitting form [1] was reached 60 min after UV exposure. In this experiment all measurements of the relative amounts of both forms were based on their fluorescence intensities. However, it is important to note that in order to induce and measure fluorescence intensity of the original and the photoconverted dye, the samples had to be exposed periodically to UV and blue exciting light. This raised a question as to the possible role of these short light exposures in further modifications of the original and the photoconverted molecules. The potential influence of these short illuminations on the production and a final concentration of the photoconverted forms can be eliminated in samples that remain and are analysed without exposure to intense light, as it is done in a mass spectrometer (Fig. 2c,d). We note that, in the MS experiments, in the absence of exposure to UV and blue exciting light, the abundance of the UV-induced photoproducts and the forms induced by exposure to hydrogen peroxide were stable for the duration of the experiment (2.5 h). This observation suggests that the forms of Hoechst 33258 that were generated by UV or H_2_O_2_ were stable in solution.

An issue to consider, when interpreting the MS experiments (Fig. 2c,d), is the fact that mass spectrometry detects the protonated forms of the dye, but does not directly provide any information about the neutral form, which is most likely the parental molecule in the UV- or oxidation-induced protonation processes. Hoechst 1+, 2+ and 3+ that are detected by MS most likely represent only a subpopulation of the whole population of the dye in the investigated solutions. Unfortunately, the relative concentrations of the protonated and the neutral form remain unknown. Another issue that needs to be taken into account is the fact that a large portion of Hoechst 33258 molecules may undergo irreversible photobleaching to a nonfluorescent form. The MS data alone do not provide the information if and which of the protonated forms are fluorescent, and whether other molecule rearrangements occur as a result of photobleaching. Some of these missing pieces of information can be extracted from analysis of MS and spectrofluorimetry data, as described below.

### Hoechst Green and Blue Fluorescence in Various pH Environments

**Fig. 3:**
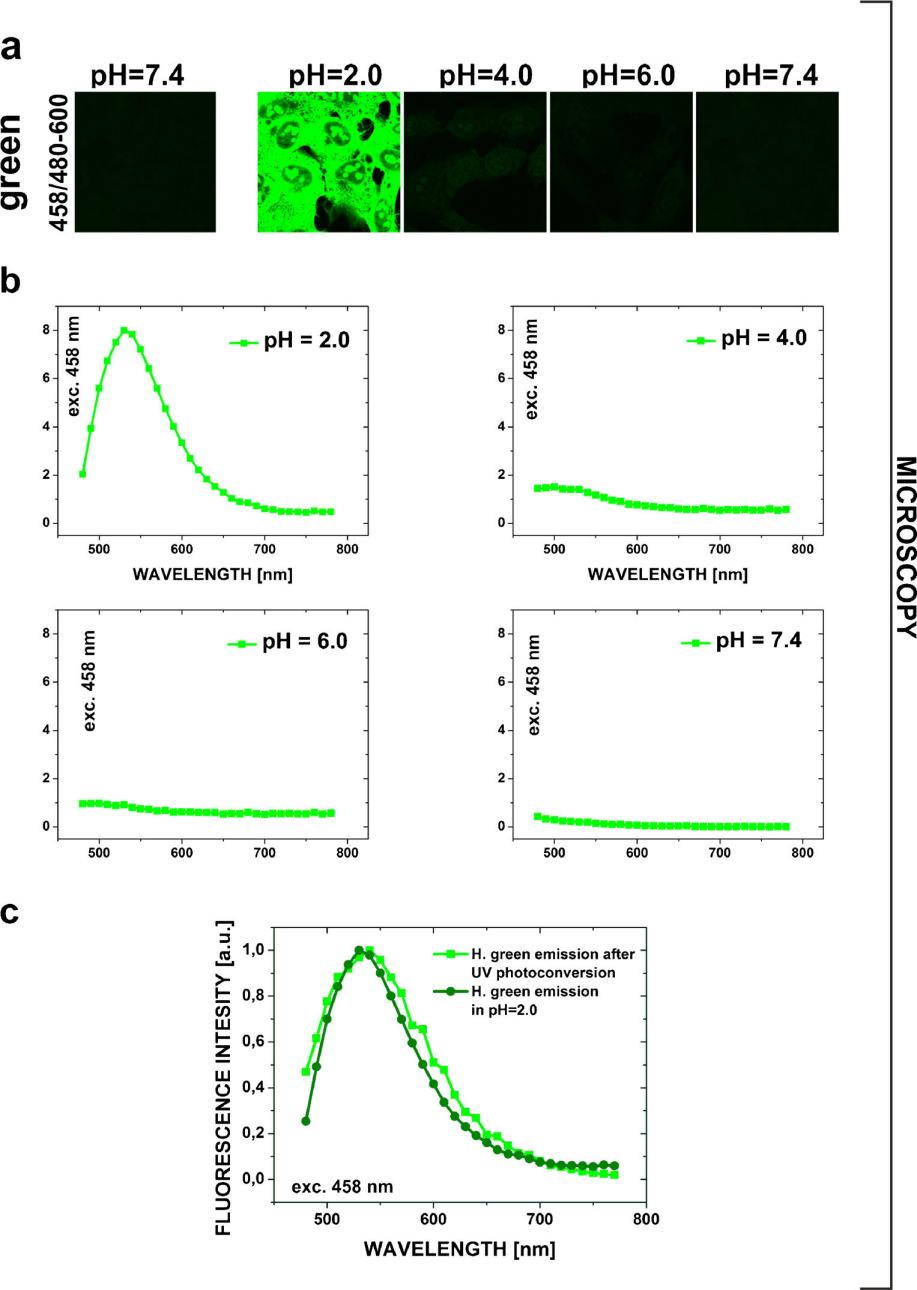
Spectral properties of the green emitting form of Hoechst 33258 generated by UV or acidic conditions. **a** – DNA-bound Hoechst 33258 green fluorescence in various pH environments. Confocal microscopy images of the green emitting form (or forms) of the dye generated after subjecting MSU 1.1 fixed cells, stained with Hoechst 33258 (2 μg/ml), to solutions of various pH (2.0, 4.0, 6.0 and 7.4). **b** – Hoechst 33258 green emission curves in various pH environments. Fluorescence spectra measured in a confocal microscope after subjecting the fixed, stained cells to solutions of various acidity (pH 2.0, 4.0, 6.0, 7.4) demonstrate a weak signal at pH 4.0 and a strong green emission at pH 2.0. **c** – DNA-bound Hoechst green emission spectra recorded after exposure to UV (*squares*), and in low pH solution (*circles*). The spectra show significant similarity

**Fig. 4:**
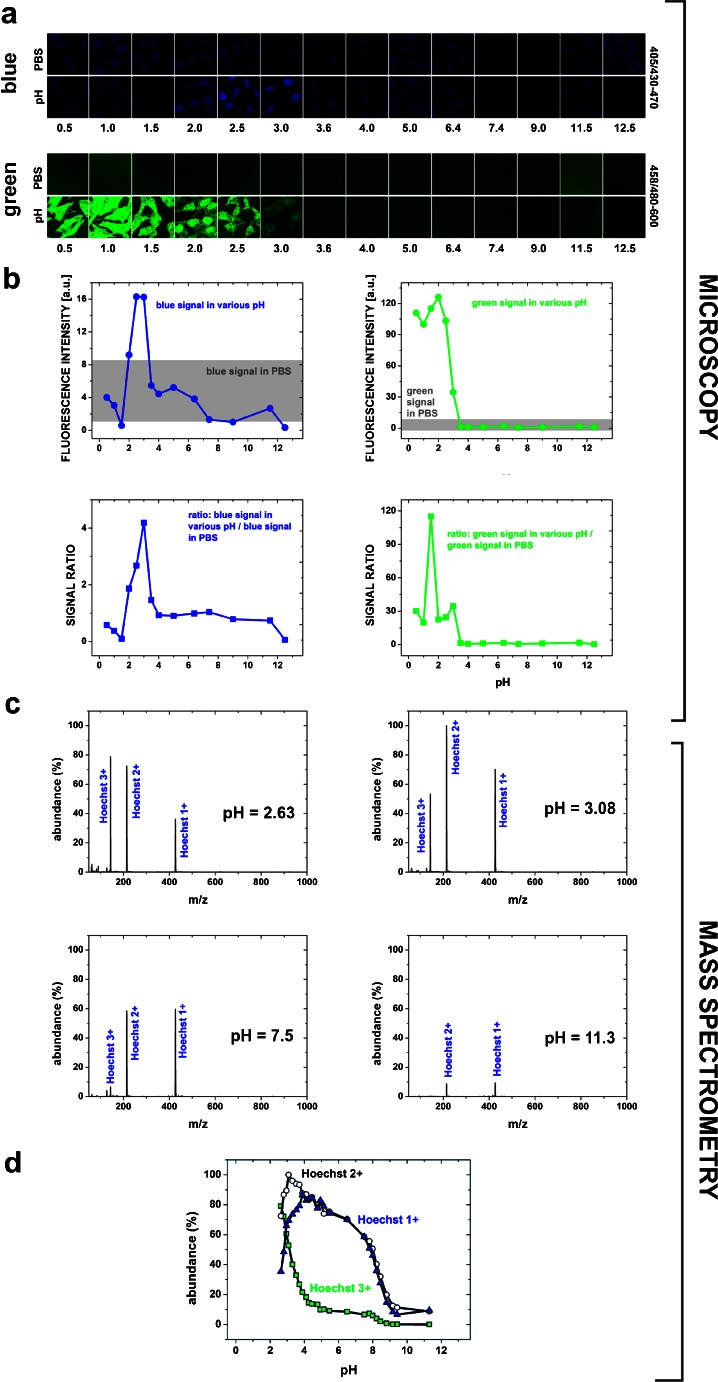
Dependence of the intensities of Hoechst 33258 blue and green fluorescence, and the abundances of the protonated forms, on acidity of the environment, analysed by fluorescence confocal microscopy and mass spectrometry. **a** – Fluorescence microscopy detection of blue and green emissions of the DNA-bound Hoechst 33258, in solutions of various pH. In both sets (*blue and green*) the top rows present control images - the blue or green fluorescence of Hoechst in PBS. The γ-function of the images presenting blue fluorescence of Hoechst was set to 0.75. The bottom rows show images of the same preparations submerged in solutions of various pH. Detection conditions for blue emission – exc. 405 nm, em. 430–470 nm; green emission: exc. 458 nm, em. 480–600 nm. **b** – Intensities of green and blue fluorescence of DNA-bound Hoechst 33258 derived from fluorescence images. The maximum intensity of the green emission is detected in pH range 0.5-2.5, while the maximum intensity of the blue emission in pH range 2.5–3.0. **c** – Mass spectrometry detection of various Hoechst 33258 protonated forms in solutions of different acidity. **d** – The abundances of various protonated forms of Hoechst 33258 in solutions of various acidity, based on mass spectrometry data

**Fig. 5:**
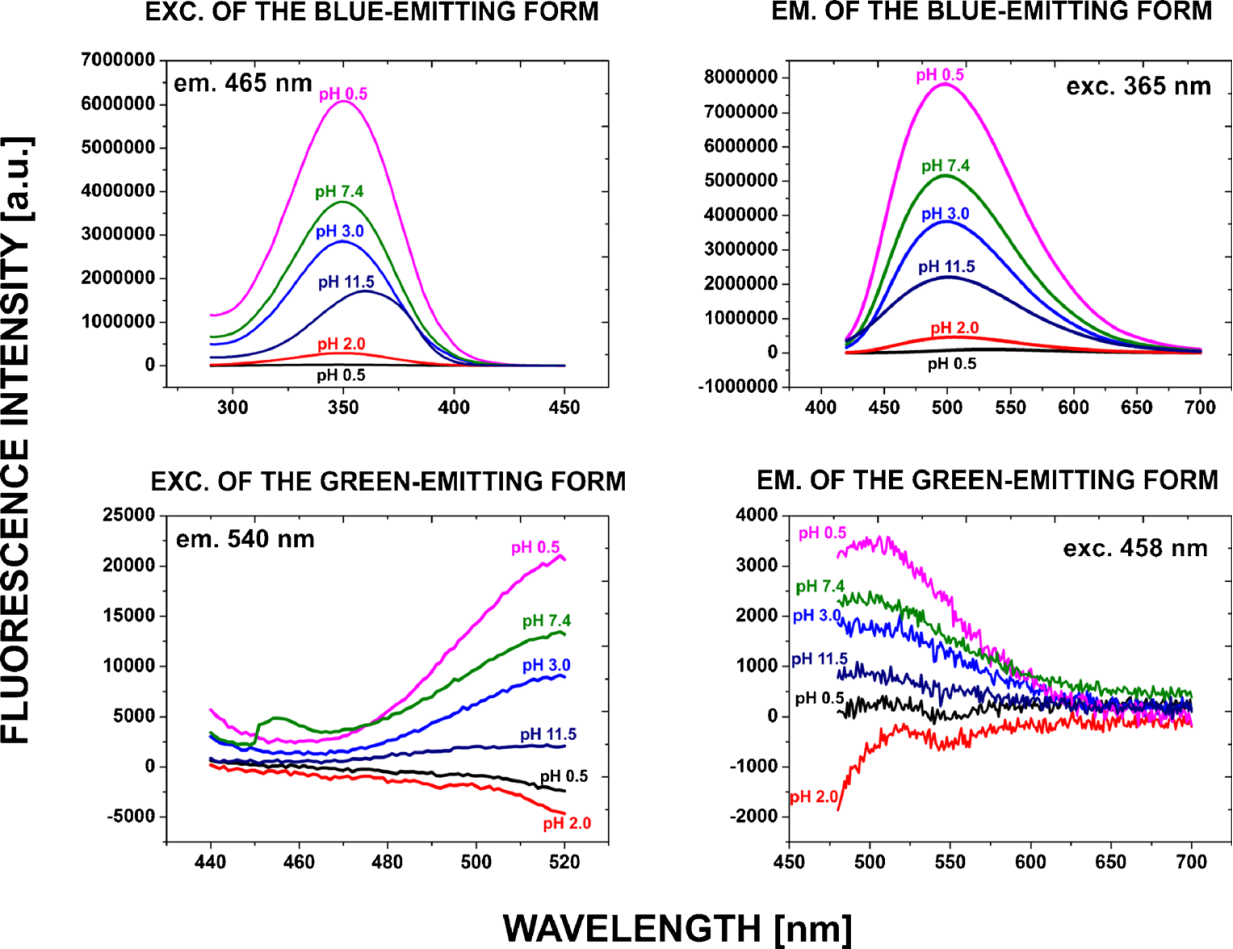
Excitation and emission spectra of the original blue-emitting form of Hoechst 33258 (10 μM) and the protonated forms of the dye in solutions of different acidity (0.5, 2.0, 3.0, 4.0, 7.4 and 11.5) measured in a spectrofluorimeter. The highest intensity of the green emission is detected at pH 4.0

**Fig. 6:**
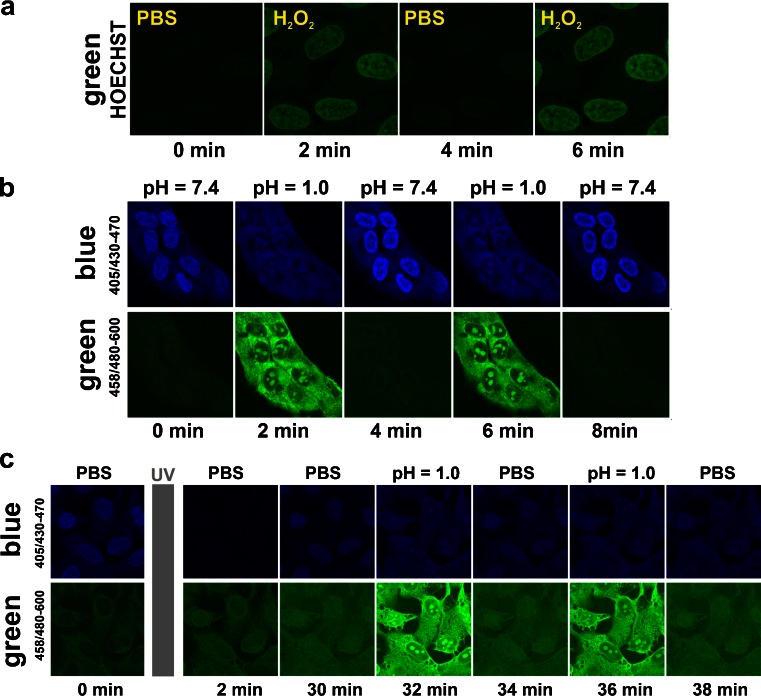
Reversibility of changes of intensities of the blue and green emissions of DNA-bound Hoechst 33258 in fixed cells. **a** – Induction and disappearance of the green fluorescence emission signal during exposure of DNA-bound Hoechst 33258 to oxidising (30 % H_2_O_2_) and nonoxidising conditions (PBS), detected by fluorescence confocal microscopy (as shown in [1], Fig. 6). The γ-function of the images was set to 0.5. **b** – Induction and disappearance of the green fluorescence emission signal during exposure of Hoechst 33258 to low (pH = 1.0) and neutral (PBS, pH 7.4) acidity environments (PBS), measured in a fluorescence confocal microscope. The γ-function of the images was set to 0.5. **c** – Acidity-induced reversible changes of the intensity of UV-induced Hoechst green fluorescence. The green emission of Hoechst was induced by UV in the same way as in the previously described experiments. Subsequently the photoconverted form of Hoechst was subjected to changes of acidity. The photoconverted, green emitting form of the dye exhibits reversible changes of the green emission, in response to changes of acidity of the environment. The γ-function of the images was set to 0.5

Blue and green fluorescence emissions of Hoechst 33258 (DNA-bound and in solution) are strongly dependent on pH (Fig. 3 and 4a,b, see also Fig. 5 and 6b, c below). Subjecting MSU 1.1 Hoechst-stained (2 μg/ml) fixed cells to environments of various pH (0.5–3.5) resulted in generation of a green fluorescence signal, which became detectable at pH 3.0, and was very prominent in a highly acidic environment (pH = 0.5–2.0) (Fig. 3a). The shapes of the emission spectra of the forms of the DNA-bound Hoechst, that were excited by blue light, were measured in a confocal microscope, in environments of pH 2.0, 4.0, 6.0 and 7.4 (Fig. 3b). No green fluorescence was detected at pH 6.0 and 7.4, but a clear green emission was detected at pH 2.0. A comparison between the green emission spectra of DNA-bound Hoechst 33258 subjected to an acidic environment (pH 2.0) and the dye exposed to UV is shown in Fig. 3c. The spectra overlap well and have their maxima at approximately 540 nm, suggesting that the form (or forms) of the DNA-bound Hoechst molecule, which is generated in a highly acidic environment, exhibits the spectral properties that are very close to the form which is induced by UV illumination of Hoechst 33258 in solution.

Further confocal microscopy and mass spectrometry measurements confirmed a very strong dependence of Hoechst 33258 blue and green fluorescence emissions on acidity of the environment (Fig. 4a-d). Confocal microscopy demonstrated that, as the pH decreased from 7.4 to 4, the intensity of fluorescence of the original blue-emitting DNA-bound form increased gradually. As pH decreased further, from 3.5 to 3.0, the intensity of blue fluorescence of the DNA-bound Hoechst increased sharply and fell again as pH decreased from 2.5 to 1.5 (Fig. 4a,b). A similar pH-dependent behaviour has been demonstrated in the case of 1+ and 2+ form of the dye by mass spectrometry, in solutions of various pH (Fig. 4c,d). Their abundance also increased as pH decreased from 7.4 to 4 (1+ form) and 3 (2+ form) and fell sharply below pH 3.0 (Fig. 4d). A comparison between the spectrofluorimetry and mass spectrometry data suggests that the 1+ form is likely the one which emits blue fluorescence. It remains unclear, however, if the 2+ form emits blue or green fluorescence. It is also important to consider that the existing body of data does not allow to exclude a possibility that the protonated forms are completely nonfluorescent and the green emitting forms have no electric charge, and are, therefore, not detected by mass spectrometry. Such a possibility seems highly unlikely, however, since very small fragments of the original Hoechst 33258 molecule would not be expected to be fluorescent and exhibit affinity to DNA. One should also note that mass spectrometry experiments provide no information about the concentrations of the parental, uncharged form of Hoechst 33258, which may also exhibit blue emission.

A different pH dependence was observed for the DNA-bound green-emitting form. Fluorescence intensity, and presumably the concentration of this form, was very weak at neutral pH and increased sharply in pH range 2.5 to 0.5 (Fig. 4a,b). In mass spectrometry data a similar pH dependence was seen for the 3+ form only. It was abundant at pH 2.5, which was the lowest pH investigated by MS. The abundance of the 3+ form was very low at neutral pH, and increased sharply when pH decreased from 3.5 to 2.5 (Fig. 4c,d). Therefore it is possible that the green emitting form of Hoechst 33258, which is induced by a high concentration of protons at pH 2.5–3.5, may be the 3+ form. It is also possible that a 4+ form is generated at pH below 2. This form might be difficult to detect in solution, in the presence of the abundant 2+ and 3+ forms, but could become readily detectable in a microscopy experiment, due to binding and accumulation in cells. Interestingly, the staining pattern at pH 0.5–2.0 differs from the typical Hoechst 33258 staining of nuclear DNA which is seen at pH 2.0–7.4. At pH 0.5–2.0 nucleoli become brightly stained, while chromatin exhibits only very weak signals (see also Fig. 6b). Thus a possibility exists that a highly protonated form of the dye which predominates in the pH range of 0.5–2.0 has an affinity to RNA. However, the abundances of various protonated forms at pH below 2.5 remain unknown. Although we conducted mass spectrometry studies at lower pH, we noticed a strong fragmentation of the dye molecule (data not shown). This phenomenon complicated interpretation of MS spectra recorded at low pH.

### Fluorescence Spectra of the Original Blue-Emitting and the Converted Green-Emitting Forms

The spectrally resolved microscopy data of DNA-bound Hoechst (Fig. 3 *and* Fig. 4a,b) were supplemented with spectrofluorimetry data of the dye solutions of different pH (Fig. 5). Intensity of the blue emission of Hoechst 33258 (we hypothesize that this emission can be attributed to the 1+ and possibly the 2+ form) in solution reached the highest value at pH 4. This was similar to microscopy measurements that showed the strongest blue emission of DNA-bound Hoechst in the buffers of pH 2.5–3.0. Note that the pH scales of microscopy and mass spectrometry experiments do not directly correspond to each other, as discussed below.

Spectral properties of the green-emitting form were more difficult to study, as demonstrated by Fig. 5. The low pH solution of Hoechst 33258 placed in a spectrofluorimeter most likely still contained all forms of the dye (0, 1+, 2+ and 3+ and possibly even the 4+), as might be inferred from the data obtained for the DNA-bound dye (Fig. 4). Thus, the putative triple-protonated green-emitting form of Hoechst 33258 was probably only a minor subpopulation of a large pool of molecules in all protonation states. It is reasonable to expect that the predominant form of Hoechst 33258, which is represented in such a solution, is still the original blue-emitting form. Thus the unprocessed emission spectrum of the solution is a convolution of emissions of all protonated forms and contains only a small contribution from the green emitting form or forms. Spectral separation of the blue and the green emitting forms is much easier in the case of a DNA-bound dye in a microscopy experiment. In this setting the photoconverted form is concentrated on nuclear DNA. Photoconversion of the DNA-bound dye is readily detectable and the blue and green emissions can be efficiently separated by using appropriate combinations of excitation wavelengths and the emission filters.

### Reversibility of the Process of Hoechst 33258 Protonation

We demonstrated previously that subjecting Hoechst 33258 to a highly oxidising environment (30 % H_2_O_2_) resulted in generation of a green-emitting form of the dye, and that this process was fully reversible [1] (Fig. 6a). Here we hypothesize that the 3+ (and possibly the 2+) protonated forms of Hoechst 33258 induced by the environment of pH below 4 might be similar or identical to the green emitting forms of the dye induced by UV and, possibly, by oxidising conditions as well. We also presume that the 4+ form might be generated at pH below 2.0. Thus, we have examined the reversibility of the protonation of Hoechst 33258 which was induced by low pH.

We subjected the DNA-bound dye to a highly acidic environment (pH = 1.0) in turns with a neutral-pH environment (pH = 7.4) (Fig. 6b). As described previously, this low pH led to generation of the green emitting form which stained nucleoli rather than DNA in chromatin. Repeating this cycle of exposures to highly acidic and neutral environments resulted in appearance and disappearance of the green emitting form of the dye, suggesting that this process (i.e. generation of the protonated forms, including the putative 4+ form) was indeed fully reversible (Fig. 6b). Moreover, when cells stained with Hoechst 33258 were illuminated with UV, using a standard mercury metal arc lamp (360/40 nm emission filter, 11 mW), the green-emitting form was generated as expected (Fig. 6c). This form stained DNA in chromatin, suggesting that this was the 3+ form. When a sample treated according to this procedure was subjected to the repeated cycles of a highly acidic (pH 1.0) and neutral environment, a concomitant appearance and disappearance of Hoechst 33258 green fluorescence in nucleoli was detected. This observation and the previously described data (Fig. 4) indicate that the UV exposure as well as pH of approximately 2 to 4 may be inducing the 2+ and the 3+ form, which have affinity to DNA, while the pH 0.5–2.0 may induce the 4+ form which exhibits affinity to RNA.

## Discussion

In the work described above we investigated generation of green-emitting forms of Hoechst 33258 (DNA-bound and in solution) by UV, oxidising conditions and by environments of low pH. In interpretation of our data we consider the 1+, 2+, 3+ and 4+ forms of the molecule. It is important to recognise, however, that the presence of the protophilic atoms of nitrogen in the molecule of Hoechst 33258 provides conditions for inducing many forms within a group embraced by a given net electric charge. For instance, the fully protonated 4+ form may actually represent a mixture of several forms from among 128 possible protonation states [3]. Also, the neutral form may actually represent a molecule with one deprotonated and one protonated nitrogen atom [2]. Thus, the various protonated forms to which we refer in the text should be understood as potentially representing one of the structures described in Fig. 1 and based on [3], or other molecular structures that also yield a given net charge.

Mass spectrometry data presented in our report indicate that UV, exposure to oxidising conditions and low pH generate protonated forms of Hoechst 33258. Under the conditions we used, UV as well as oxidation appeared to induce 1+, 2+ and 3+ forms. Acidity led to generation of 1+, 2+, 3+ and possibly 4+ form as well. The protonation states that we observed were dependent on pH as would be expected.

MS and spectrally resolved data describing Hoechst 33258 in various pH environments were gathered in order to provide more information about the chemical nature of the green-emitting form (or forms) of the dye. This approach was based on an assumption that not only the parental molecule of the dye, but also the protonated forms of Hoechst 33258 emit fluorescence. In principle one should also consider a possibility of the protonated forms being nonfluorescent. In this case any attempt to link fluorescence changes (the observed shift to longer wavelength) with the protonation states would have no ground. As mentioned before, such a scenario is unlikely, since one would have to assume that all the protonated forms of Hoechst 33258 are nonfluorescent, while some other forms that carry no electric charge and are undetectable by MS would exhibit blue and green fluorescence. Such a possibility is highly improbable. Thus, in our reasoning we assume that the protonated forms are indeed fluorescent, and their spectral properties depend on their protonation state.

A comparison between the pH-dependent changes of abundances of various protonation forms of Hoechst 33258, as demonstrated by MS, and changes of fluorescence properties measured with spectrally resolved microscopy and spectrofluorimetry, leads us to postulate that the 1+ form emits blue fluorescence, while the green-emitting form induced by UV or low pH is the 3+ form (and possibly the 4+ form as well). This reasoning is based on the observation that the abundance of 3+ form detected by MS, and the intensity of fluorescence of the green form detected by spectrally resolved microscopy, increase rapidly as pH falls from approximately 4 to 2. The ranges of pH at which the increases occur are similar, however we note that the local pH in the immediate vicinity of DNA may differ slightly from the pH in the bulk of the buffer. This putative difference may mean that the DNA-bound Hoechst reside in an environment of somewhat different pH than the bulk solution i.e. different than the value given on the axis of the graph in Fig. 4. Moreover, it is not known if any quenching of fluorescence occurs in the case of DNA-bound Hoechst, and how acidity influences this process and the overall detected fluorescence intensity. While the green fluorescence can be attributed to the 3+ form with fair confidence, unfortunately, the available MS and fluorimetry data are not sufficient to assign blue or green emission to the 2+ form.

It is possible that the 4+ form was induced at pH below 2. MS studies of Hoechst 33258 in such a highly acidic environment were complicated by fragmentation of the molecule. Moreover, according to Ladinig et al. 2005 [3] the 4+ form of Hoechst becomes significantly represented only at pH < 0. Therefore, in order to visualise it on MS spectra it is necessary to generate a very high concentration of this form in solution, which is technically cumbersome. In contrast, microscopy visualisation of the 4+ form is easier due to a high local concentration of this form bound to nucleic acids, and an ability to spectrally separate green and blue emissions using carefully selected excitation wavelengths and emission filters. Microscopic spectrally resolved data indicate that the form of the dye which is present at pH 0.5–2.0, that is a putative 4+ form, exhibited affinity to RNA. Conspicuously, the nucleoli of the fixed cells were brightly stained (green emission), while the areas rich in DNA emitted only very weak fluorescence. In this context it is important to mention that Hoechst 33258, as most if not all fluorescent probes used for labelling DNA, exhibits also some affinity for RNA [12]. Thus, the putative 4+ form may differ from 1+, 2+ and 3+ forms by having a higher affinity to RNA than DNA. Such a phenomenon, although unexpected, agrees with the fact that small modifications of the bis-benzimidazole molecule have been shown to result in strong changes of affinity and mode of binding to DNA [13, 14]. Interestingly, the issue of a relationship between protonation of Hoechst 33258 and a conformation of the molecule appears to remain unresolved, since minor as well as substantial conformation changes were postulated [2]. There is another factor which may have contributed to the nucleolar staining we observed. It is possible that under the acidic conditions self-quenching of fluorescence of Hoechst bound to DNA becomes very efficient, while quenching on RNA is poor. Such a phenomenon could contribute to the pattern of staining observed at pH below 2. More experimental work is required to resolve this issue. Another factor whose role is difficult to assess is denaturation of DNA at low pH, potentiated by exposure to exciting light [15]. However, we postulate that this phenomenon was not responsible for the staining pattern we observed, since acid denaturation would be expected to generate single stranded DNA. Such denatured DNA might be expected to be stained green, as RNA in nucleoli, at low pH.

Our observation of changes of the blue fluorescence emission accompanying protonation are in agreement with the previous reports, suggesting that changes in the chemical structure of Hoechst 33258 result in significant changes of the electronic charge distribution and, as a consequence, the spectral properties of the dye [5]. Also the data reported here are consistent with the view that the predominant form of Hoechst 33258 in a neutral pH is the single-protonated form, while in a low pH environment it is the triple-protonated form [3], and at pH 4.5 Hoechst 33258 exists in a double-protonated form. Hoechst 33258 fluorescence emission spectra, excited by 365 nm, recorded at four different pH values (7, 4.5, 1.5 and 11) were reported before [5]. It is reasonable to expect that at this excitation only the emission component which we briefly refer to as ‘blue’ was detected. This study showed that lowering the pH value from 7 to 4.5 resulted in a significant increase in fluorescence yield of Hoechst 33258 and in a red-shift of the emission spectrum by ~22 nm. Presumably this shift was associated with generation of the 3+ protonated form of the molecule. Further lowering of the solution pH did not result in any further increase, but on the contrary, it led to a decrease of the fluorescence intensity. We have also seen an increase in the blue fluorescence of Hoechst in solution treated with solution of pH value 4.0 (Fig. 5) and Hoechst 33258 on DNA in the pH value of 3.0 (Fig. 4), nevertheless we have not noticed any increase of the blue (or green) fluorescence of Hoechst in a strongly alkaline environment. Mass spectrometry showed that in a strongly alkaline environment (pH ~11.0) Hoechst 33258 underwent strong ionisation with no prominent peaks of the protonated forms visible on MS-spectra (data not shown). Microscopy and spectrofluorimetry also confirmed that when Hoechst was subjected to a highly alkaline environment its blue fluorescence was significantly decreased (Figs. 4 and 5).

The dependence of the intensity of Hoechst 33258 blue fluorescence on pH was also reported by Görner and collaborators [16]. These experiments showed that Hoechst reached the highest intensity of the blue fluorescence around pH value 4.0. In our experiments the highest blue fluorescence intensity of Hoechst 33258 was observed near pH value of approximately 3.0 (Fig. 4). This difference may arise from the fact that in our experiments DNA-bound Hoechst was investigated while the data in the report [16] describe measurements of Hoechst 33258 in solution.

## Conclusions

Upon exposure to UV DNA-bound Hoechst 33258 undergoes photoconversion to a blue-excited green-emitting form. Mass spectrometry revealed that UV, as well as exposure to oxidising conditions, result in generation of the protonated (1+, 2+, 3+ and possibly the 4+) forms of the dye. While the 1+, 2+ and 3+ forms of the dye exhibit affinity to DNA (fluorescence of nuclear DNA is strong, while RNA is very weak), the protonated form which exists in pH 0.5 (presumably the 4+) exhibits affinity to RNA.
